# Adipose Tissue Dendritic Cells: Critical Regulators of Obesity-Induced Inflammation and Insulin Resistance

**DOI:** 10.3390/ijms22168666

**Published:** 2021-08-12

**Authors:** Shindy Soedono, Kae Won Cho

**Affiliations:** 1 Department of Integrated Biomedical Science, Soonchunhyang University, Cheonan 31151, Korea; shindysoedono@sch.ac.kr; 2 Soonchunhyang Institute of Medi-Bio Science (SIMS), Soonchunhyang University, Cheonan 31151, Korea

**Keywords:** obesity, adipose tissue, dendritic cells, insulin resistance, inflammation, antigen presentation

## Abstract

Chronic inflammation of the adipose tissue (AT) is a critical component of obesity-induced insulin resistance and type 2 diabetes. Adipose tissue immune cells, including AT macrophages (ATMs), AT dendritic cells (ATDCs), and T cells, are dynamically regulated by obesity and participate in obesity-induced inflammation. Among AT resident immune cells, ATDCs are master immune regulators and engage in crosstalk with various immune cells to initiate and regulate immune responses. However, due to confounding markers and lack of animal models, their exact role and contribution to the initiation and maintenance of AT inflammation and insulin resistance have not been clearly elucidated. This paper reviews the current understanding of ATDCs and their role in obesity-induced AT inflammation. We also provide the potential mechanisms by which ATDCs regulate AT inflammation and insulin resistance in obesity. Finally, this review offers perspectives on ways to better dissect the distinct functions and contributions of ATDCs to obesity.

## 1. Introduction

Obesity is a chronic low-grade inflammatory state, essential in the pathogenesis of metabolic disorders, including insulin resistance and type 2 diabetes [1]. The adipose tissue (AT) is the primary and first organ affected by inflammation and sustains obesity-induced inflammation [2]. Various immune cell types, including macrophages, dendritic cells (DCs), and T and B cells, have been identified in the AT and implicated as key players in obesity-associated immune responses. Numerous studies demonstrated that the composition of both myeloid and lymphoid cell profiles dynamically changes, and their inflammatory output shifts with increased adiposity [3,4]. In addition to the specific immune cell population changes, crosstalk between innate and adaptive cells is crucial in the regulation of inflammation. Therefore, understanding AT inflammation through the integration of innate and adaptive immune cells will provide a new understanding of the pathophysiology of diabetes and related metabolic syndromes.

Dendritic cells are the latest discovered hematopoietic stem cell lineage and are classified as part of the innate immune system. Their main role is to act as antigen-presenting cells (APCs) and as such have the capacity of recognizing pathogens, become activated through antigen processing, migrating, and the presenting of the antigen to naïve T cells, as well as the secreting of cytokines [5,6,7]. Since DCs control innate and adaptive immunity, therefore orchestrating immunity, they have been considered as key regulators in obesity-induced inflammation [8,9,10,11]. However, AT macrophages (ATMs) also express CD11c, a DC marker, which makes it difficult to distinguish AT dendritic cells (ATDCs) from ATMs. Moreover, not only DCs, but macrophages can also function as APCs that respond to local environmental cues, adapting their morphological and functional phenotype to these signals. They regulate the types of adaptive immune response initiated by obesity. Thus, the understanding of ATDC’s specific function has been limited by confounding markers. 

Several research groups, including us, have explored new markers to delineate ATDCs. Our group previously reported that CD64 is exclusively expressed in ATMs, and it can be used to distinguish ATMs from ATDCs in both obese human and mouse [12]. Using CD64 and CD11c, we identified ATDCs as an independent contributor to AT inflammation and insulin resistance in obesity [13]. Recently, several studies revealed new DC functions and expanded the understanding of ATDCs in obesity. In this review, we summarize the characteristics of DCs, especially in the AT. Furthermore, this review describes the role of ATDCs in both lean and obese states. Finally, we provide the potential regulatory mechanisms of ATDCs as the key players in obesity-induced inflammation and insulin resistance.

## 2. Phenotypic Characteristics of Adipose Tissue Dendritic Cells

### 2.1. Dendritic Cells: Subsets and Properties

Dendritic cells are professional APCs, defined by a high expression of integrin alpha X (ITGAX) or CD11c as well as major histocompatibility complex-II (MHC-II). Under steady state, there are two main DC lineages in blood and tissues, both originating from the bone marrow hematopoietic stem and progenitor cells (HSPCs): the comparatively rare plasmacytoid DC (pDC), and the more common conventional DC (cDC) [14]. While pDCs are major regulators in antiviral CD8^+^ T cell activation [15], pDCs show lower-level expression of genes such as CD11c and MHC-II and poor ability to stimulate T cells [16,17]. Thus, in tissues, cDC is the major resident DC capable of antigen presentation (AP), as well as cytokine and chemokine production for pathogen elimination [14,18]. Under inflammatory conditions, monocyte-derived DCs (moDCs) arise from monocyte recruitment with the same phenotype as cDCs [19].

Conventional DCs can be further categorized into two subsets based on different lineage markers and transcriptomic profiles: cDC1 (CD4^−^ CD8α^+^ CD103^+^ CD205^+^ CD11b^−^ CLEC9A^+^ XCR1^+^ CD24^+^) [16,19,20,21,22] and cDC2 (CD4^+/−^ CD8α^−^ CD205^−^ CD11b^+^ CLEC4A4^+^ CD24^+^) [23,24,25]. cDC1s have a higher expression of transcription factors interferon regulatory 8 (IRF8) and BATF3, while cDC2s are regulated by interferon regulatory 4 (IRF4) [13,16,24]. cDC1 specialize in cross-presentation to CD8^+^ T cells and cDC2 activate CD4^+^ T cell differentiation. Homolog subsets and function in mouse DCs can be found in human DCs with different surface marker, which are categorized into three populations: cDC2 (CD1c^+^), cDC1 (CD141^+^), and pDC (CD303^+^). In addition, human moDCs expressed CD14^+^, presumed to be the precursor of inflammatory DCs [26]. Despite marker differences, functional properties and distribution of each DC subset among species are similar. In steady state, the population of cDC1 is lower than that of cDC2. Further, visualization of cDC subsets reveals differential localization in lymphoid tissues. In the lymph node (LN) and the spleen, cDC1s are located in the T cell zone. On the contrary, cDC2s are more peripherally distributed and express various receptors to respond to antigens [23,24,27,28,29]. Whether the localization of cDC subsets in peripheral tissues, including the AT, would be different remains elusive.

### 2.2. ATDC Characteristics, Quantitation, and Subsets in Lean and Obese States

The identification and characterization of ATDCs have been extensively studied in many experimental mouse models using a distinct set of cell-surface markers, including CD11c. Bertola et al. [30] demonstrated that CD11c^high^B220^−^ cells are a heterogeneous group of DCs in the AT that contain several distinct subpopulations, including CD11c^+^F4/80^neg^ and CD11c^+^F4/80^low^. At the same time, another group reported that substantial proportions of CD11c^+^ cells in AT are DCs, which was confirmed in a DC deficient model, *Flt3l^–/–^* mice [31]. The existence of ATDCs was also supported by the observation of some CD11c^+^ cells displaying a rough surface with multiple pseudopodia, identical to the typical DC morphology [30,32]. Moreover, these cells can induce T cell polarization from naïve T cells [12,30,33], which confirms the existence of functional ATDCs in the lean state to maintain a tolerogenic state. 

Similar to other peripheral tissue DCs, ATDCs can be defined by a high expression of CD11c and MHC-II. Around 80–90% of ATDCs express CD11b, indicating that cDC2 is the predominant ATDC subset. Co-stimulatory molecules, such as CD40, CD80, and CD86, are also expressed by ATDCs. However, these surface antigens are confounding markers to define pure ATDCs, since ATMs, the most abundant myeloid cells, also express them to different extents depending on inflammatory status [30,34,35]. Previously, we reported that separation of ATDCs from ATMs can be achieved by using CD64 and MerTK, as ATDCs do not express those markers [12,36]. The finding of CD11c^+^CD64^−^ as putative ATDCs, independent of ATMs contamination, makes it possible to distinguish ATDCs from ATMs in obesity. In addition to those markers, the transcription factor ZBTB46 has been known to be useful to define cDCs, but not pDCs, macrophages, or monocyte-derived cells [37]. Consistently, gene expression array data showed that *Zbtb46* is exclusively expressed in ATDCs, both in lean and obese AT [12]. Visualization of cDC using a reporter mouse (*Zbtb46*-GFP) showed that only 40% of cells among CD11c^+^MHC-II^+^ cells are GFP^+^ cDC subsets in visceral adipose tissue (VAT), indicating the usefulness of ZBTB46 as a marker to delineate ATDC subset characteristics [38].

ATDCs have been recognized as a substantial proportion of infiltrating cells within the AT [39]. Accumulation of DC in subcutaneous AT (SAT) of obese human has been reported using marker CD11c^+^ CD1c^+^, the increment of which was correlated with body mass index (BMI) [30]. In mouse studies, infiltrating ATDCs are associated with crown-like structures (CLS) in the AT of high fat diet (HFD)-fed mice [12] and appear to accumulate with moderate HFD exposure [12,31]. In VAT, ATDCs have been shown to significantly increase after diet-induced obesity (DIO), from <5% in normal diet (ND) to around 10% after 8 weeks of HFD [12,40]. Based on flow cytometry of obese ATDCs, this increasing population is mainly coming from CD11b^+^ subsets or cDC2s [12]. Sharing markers with cDCs, moDC recruitment is increased in obese AT [41,42]. It is also reported that obesity increases the pDC (CD11c^+^PDCA1^+^ cells) population in VAT [31]. Collectively, despite the differences among ATDC subsets, all subsets in obese VAT were increased compared to lean VAT [12,31]. Interestingly, recent single cell analysis revealed the existence of three different ATDC populations in obese VAT: MHC-I presenting DCs, MHC-II presenting DCs, and replicating DCs or moDCs, whose proportions were changed by obesity [43]. Advances in single cell multiomics will provide a more comprehensive understanding of ATDCs in obesity.

Immature and mature DCs have different phenotypic and functional characteristics [32]. During maturation, DCs go through phenotypic alterations, such as induction of genes related to AP. In parallel with inflammation, obesity upregulates the surface presentation of MHC molecules in ATDC, indicating a higher AP. Expression of co-stimulatory molecules such as CD40, CD80, and CD86 was also enhanced in obese ATDCs [12,30,44]. A wide array of cytokines produced by DCs are also controlled by obesity. Gene expression data of sorted ATDCs demonstrated that obese ATDCs show a higher expression of IL-12, IL-18, and IL-6, indicating that obesity induces ATDC activation and maturation [12,30]. These alterations, including AP properties and cytokine production, would induce the different functional properties of ATDCs in obesity.

### 2.3. Mechanism of Obesity-Induced ATDC Expansion

The accumulation of immune cells in peripheral tissues is tightly regulated by the local proliferation, migration from blood, or both. In early stages of obesity, ATDC proliferation, as detected by the Ki67 marker, is induced in both VAT and SAT as early as three days after HFD initiation, and the number of ATDCs significantly increases after seven days of HFD, both in VAT and SAT. Furthermore, Ki67^+^ ATDCs were found to keep increasing until 14 days of HFD in SAT. In contrast to SAT, ATDC proliferation is not positively correlated with the frequency of ATDCs in VAT [40]. Increased infiltration rates of ATDCs to VAT is in accordance with chronic inflammation progression, where it keeps increasing after 4 weeks HFD and goes up further at least until 8 weeks of HFD [12,40]. However, the ATDC population percentage is reduced after 16 weeks of HFD, specifically in VAT but not in SAT [12]. These results suggest that mechanisms responsible for the obesity-induced ATDC expansion may be AT depot-specific. It still remains to be investigated whether ATDCs in VAT and SAT have different features, functions, and/or specific ones during obesity progression.

Blood contains DC precursors and differentiated DC subsets as well as pluripotent HSPCs, which are able to differentiate into different DC subsets and circulating monocytes. Circulating DCs and their precursors exit the blood in response to tissue-specific recruitment signals, like a response to inflammation or as part of tissue-resident DC turnover [45]. During obesity, ATDC migration tracking has been studied using *Ccr7*^−/−^ mice, as ATDC migration is specifically dependent on CCR7 but independent of CCR2, whereas monocyte-derived ATMs are dependent on CCR2. This study also confirmed parallel mechanism of DC migration in human, which was CCR7-dependent [12]. Although chemokine-chemokine receptor pathways such as CCR2-CCL2, CCR5-CCL5, and CCR6-CCL20 are important to gain access to non-lymphoid peripheral tissues [45], there are limited studies identifying specific chemokines regulating ATDC accumulation and arrest in tissues. It remains to be investigated how excess nutrition in early obesity triggers the increase of ATDC populations and whether it is caused by ATDC’s maturation and migration signal itself or other cell-derived factors.

## 3. Functional Characteristics of ATDCs

### 3.1. Body Weight Regulator

Inflammation regulates energy metabolism and is directly involved in the pathogenesis of obesity-induced metabolic disorders. As a contributor to inflammation, DCs are emerging as an important regulator of energy homeostasis as well. Apparently, the loss of DCs in a mouse model (*Flt3l*^−/−^ mice) prevents HFD-induced weight gain [31]. FMS-like tyrosine kinase 3 (Flt3) is expressed by early hematopoietic progenitor cells, and without its ligand (Flt3L), there is a defect in migration and expansion from bone marrow to the peripheral lymphoid tissue [46], causing DC ablation in peripheral tissues. Interestingly, DC depletion in *Flt3l*^−/−^ mice resulted in resistance to obesity and normal body weight gain. Moreover, *Flt3l^−/−^* mice showed increased heat production, indicating increased metabolic rate as a potential mechanism to prevent body weight gain [31].

Another study to characterize DC function was performed in the *Csf2*^−/−^ mouse model, another whole-body DC depletion model. Granulocyte-Macrophage Colony Stimulating Factor (GM-CSF) is encoded by *Csf2* and is essential for moDC generation, DC maturation, and DC survival, to become fully functional as APCs [47]. In *Csf2*^−/−^ mice, reduction of DCs (CD45^+^ CD11b^+^ CD11c^+^ MHC-II^+^ F4/80^low^ cells) is followed by lower body weight compared to *Csf2^+/+^* mice [47,48,49]. *Ccr7^−/−^* mice also lack peripheral DCs, as lacking CCR7 expression lowers the ability of DCs to migrate in response to their activation. Interestingly, *Ccr7^−/−^* mice are protected against body weight gain under an HFD challenge, with enhanced energy expenditure and *Ucp1* activation in VAT and brown AT (BAT) [50].

Overall, these studies demonstrate the body weight alteration when most of DCs disappear. It would be interesting whether the effect of global depletion of DCs in the various knockout models is a direct or indirect effect on the reduced body weight. Another interesting question is whether antigen-induced activation might be involved in ATDC’s function. However, the results from the above whole-body DC depletion models do not exclude the possibility of the developmental defects as well as the interference of other cells. In *Flt3l^−/−^* mice, the development of other cells regulated by DCs was also altered, including natural killer (NK) cells, regulatory T cells (Tregs), and B cells [51]. Similarly, in *Csf2^−/−^* mice, GM-CSF regulates the development of granulocytes as well as monocytes [48], whereas in *Ccr7^−/−^* mice, CCR7 is also expressed by certain T and B cells [50]. Thus, further studies are required to clarify if DC is the real regulator of body weight and if yes, how DC directly or indirectly affects body weight, whether through DC’s derived factor or DC’s AP function.

### 3.2. Regulator of Adipose Tissue Homeostasis in the Lean State

In steady state, DCs play an important role in tissue homeostasis by maintaining the peripheral tolerance. Tolerogenic function of ATDCs in the lean state may be related with immature phenotype, which is characterized by a lower extent of maturation marker expression such as CD80 and CD86 [52]. It is also known that tolerogenic functions of DCs can be directed and enhanced by the targeted delivery of defined antigen. The functional characteristics of ATDCs in the lean state could be partly observed in the inducible CD11c-DTR mouse model, which expressed human diphtheria toxin receptor (DTR) under *Cd11c* promoter [53]. After diphtheria toxin (DT) administration in lean mice, depletion of CD11c^+^ cells did not disrupt CD4^+^ T cell numbers and proliferation [12,54], suggesting steady-state CD4^+^ T cell activation. A similar model using MHC-II^fl/fl^ CD11c-Cre (M11cKO) showed no differences in metabolic profiles in the lean state, even though MHC-II expression was depleted in CD11c^+^ cells [33]. This might be due to the heterogeneity of ATDC populations, containing several distinct subpopulations which could replace each other’s functions.

ATDCs’ tolerogenic function is properly maintained by intercommunication with adipocytes. Macdougall et al. found that each cDC subset contributes to a tolerogenic environment by different mechanisms. The cDC1 subset has an active Wnt/β-catenin pathway, whereas the cDC2 subset has an active PPARγ pathway that negatively regulates inflammatory gene expression through transrepression of NF-κβ target genes. In the lean state, adipocyte-derived β-catenin would induce activation of the Wnt/β-catenin signaling pathway in cDC1 and further induce anti-inflammatory cytokine IL-10 production. Separately, adipocyte-secreted dietary lipids could induce PPARγ signaling in cDC2, which would suppress its activation of inflammatory DC. Induction of both signaling pathways could suppress toll-like receptor-4 (TLR4)-induced inflammation in VAT; however, interestingly, cDC1 tolerogenic function deletion by *Ctnntb1*^−/−^ (*Ctnnb1^fl^*^/*fl*^ zDC-Cre) and cDC2 by *PPARγ*^−/−^ (*PPARγ^fl^*^/*fl*^ zDC-Cre) in ND-fed mice did not produce any differences in metabolic parameters [38]. 

A specific tolerogenic DC subset, called perf-DC, was characterized by Zangi et al. by its ability to secrete perforin. The perf-DC subset is derived from HSPCs (defined as Lin^−^ Sca^+^ cKit^+^) and contributes to 2–4% of CD11c^+^ DC within the secondary lymphoid organ (SLO), which further increases after GM-CSF administration. The unique perf-DC tolerogenic function was shown by the specific interaction between peptide-MHC and the T-cell receptor (TCR) of CD8^+^ T cells, which would activate toll-like receptor-7 (TLR7) and triggering receptor expressed on myeloid cells-1 (TREM-1) signaling in DC to induce perforin and granzyme A secretion, which could selectively kill CD8^+^ T cells [55]. Further, in mice with depletion of CD11c^+^ Perf^+^ DC (*Itgax*-DTA-*Prf1*^−/−^) under steady state, long-term metabolic alterations similar to type 2 diabetes were produced. Even under ND, five months after depletion, it still resulted in increased body weight, body fat, leptin level, TNFα level, glucose intolerance, insulin resistance, adipocyte hypertrophy, and CLS formation [56]. Collectively, in the lean state, certain ATDC subsets would play important roles in AT homeostasis, by acting as a gatekeeper to control adaptive immunity as well as nurture an anti-inflammatory environment. 

### 3.3. Key Regulator of Adipose Tissue Inflammation in Obesity

The functional characteristics of ATDCs in obesity have been established using various loss-of-function models. Whole-body DC depletion models such as the *Flt3l*^−/−^ mice and *Csf2*^−/−^ mice showed protection from obesity-induced insulin resistance, indicating the role of DCs in obesity-induced glucose homeostasis [31,48,49]. While *Flt3l*^−/−^ mice are resistant to DIO [31], *Csf2*^−/−^ mice exhibit insulin sensitivity despite increased adiposity in response to HFD [48,49]. These discrepancies imply diverse protective mechanisms of DC against obesity-induced glucose homeostasis. Another model to characterize DC function is the *Ccr7*^−/−^ mouse, which contains few peripheral DCs due to inhibition of ATDC chemotaxis [12]. Interestingly, *Ccr7*^−/−^ mice exposed to HFD did not suffer ATDC accumulation in VAT followed by protection to AT inflammation and insulin resistance, indicating the important participation of ATDC migration in early stages of obesity [12,50]. Improved metabolic profiles were accompanied by reduced CLS formation, lower *Il6*, and higher *Foxp3* expression in VAT [12]. Overall, these studies showed that DCs in the AT could be an important regulator of glucose homeostasis, related to AT homeostasis during obesity-induced inflammation.

To avoid any developmental defect and delineate the ATDC function in the obese state, an inducible DC ablation model has been considered. Exploration of CD11c^+^ cell function during obesity, including ATDC and CD11c^+^ ATMs, has been observed in CD11c-DTR mice. Ablation of CD11c^+^ cells in obese mice lead to the improvement of obesity-induced inflammation and insulin resistance, accompanied by reduction of T cell activation, with higher proliferation of Tregs but not conventional T cells [57]. Another study with same model, showed that CD11c^+^ cell ablation results in the reduction of CLS formation with increased *Il10* and decreased *Il6* and *Mcp1* expression in VAT [54].These results indicate the crucial role of ATDCs to activate CD4^+^ T cells that contributes to AT inflammation in obesity.

ATDCs are heterogeneous groups composed of various subpopulations. Although it is known that the CD11c^+^ ATDC is the key player in obesity, the functions of the specific ATDC subset were recently identified. Macdougall et al. reported that deletion of tolerogenic function in each specific cDC subset, cDC1 and cDC2, using *Ctnntb1*^−/−^ mice and *PPARγ*^−/−^ mice, respectively, resulted in worsened insulin resistance under 12 weeks HFD [38]. Perf-DC was identified as the distinct tolerogenic subset of ATDCs in obesity by using *Itgax*-DTA-*Prf1*^−/−^ chimera HFD mice, where CD11c^+^ Perf^+^ DC depletion exacerbates metabolic parameters such as body weight, VAT weight, liver weight, liver triglycerides, leptin level, insulin level, and also proinflammatory cytokine secretion, including IL-1β and TNFα [56]. Overall, these data implied that the tolerogenic function of certain DC subsets could be an important contributor in AT inflammation. 

The AP function of ATDCs is considered the primary pathway that regulates obesity-induced inflammation. A number of studies showed that deletion of AP genes such as MHC-II, CD40L, and CD80/CD86 lead to marked changes in obesity-induced inflammation and insulin resistance [58,59,60,61,62]. However, since AP can also be performed by other APCs in addition to ATDCs [57], a direct and specific ATDC depletion model should be considered to elucidate the AP function of ATDCs in obesity. To this end, Lumeng groups developed MHC-II^fl/fl^ Itgax-Cre (M11cKO) and MHC-II^fl/fl^ Lysm-Cre (MMKO) mice as putative ATDC- and ATM-specific MHC-II knockout models [12,33]. Regarding the obesity-induced AT inflammation, both M11cKO mice and MMKO mice showed decreased CD4^+^ T cells. The reduction of TCR expression and lower frequency of effector/memory CD4^+^ T cells were also observed in M11cKO and MMKO mice, respectively. These results support the existence and the importance of APC function of ATDCs in obesity-induced AT inflammation. 

**Figure 1:**
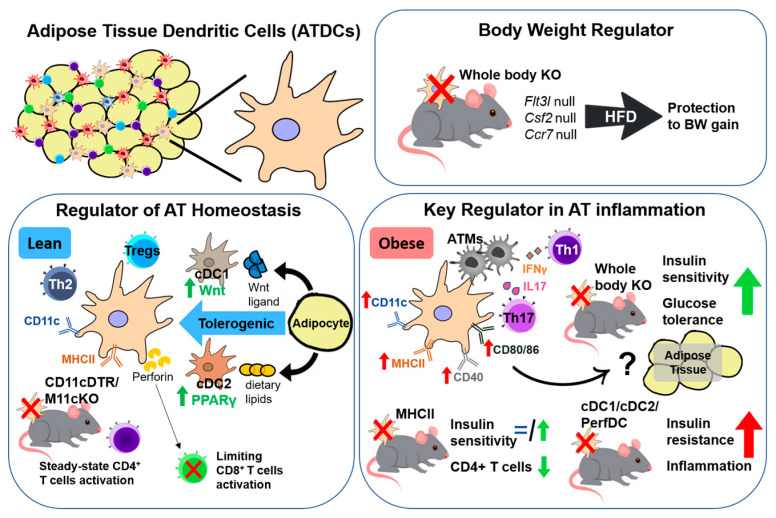
Functional characteristics of adipose tissue dendritic cells (ATDCs). In steady state, ATDCs have a tolerogenic function (green arrow) and together with adipocytes maintain adipose tissue (AT) homeostasis. Obesity challenges in the absence of dendritic cells (DCs) prevent body weight (BW) gain and become a key regulator in AT inflammation and insulin resistance (red arrow) by interacting with T cells and adipose tissue macrophages (ATMs).

Taken all together, the ATDC is a key player in obesity-induced inflammation and insulin resistance, where it potentially regulates body weight while preserving AT function and homeostasis, especially in obesity (Figure 1). However, the contribution of ATDCs is still not fully explored. The characterization of the AP function of specific ATDC subsets and the MHC-I presentation of ATDCs is also warranted. 

## 4. Mechanisms of ATDCs in Obesity-Induced Inflammation and Insulin Resistance

**Figure 2:**
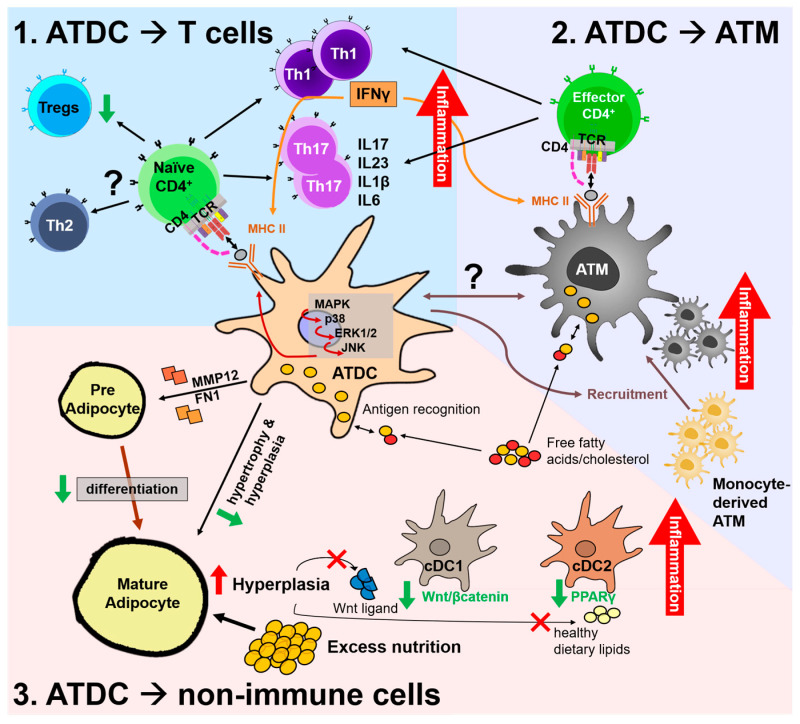
Potential regulatory mechanisms by adipose tissue dendritic cells (ATDCs) in the adipose tissue (AT) environment by interaction with T cells (**1**), AT macrophages (ATMs) (**2**), and non-immune cells (**3**). In obese AT, uptake of antigens or lipids such as free fatty acids and/or cholesterol by ATDCs induces activation of the mitogen-activated protein kinase (MAPK) pathway, leading to increased MHC-II expression and induced maturation. 1. Obese ATDCs lead the Th1 and Th17 subset proliferation that creates the pro-inflammatory environment. Released IFNγ is capable of inducing MHC-II expression in antigen-presenting cells, creating a loop to amplify Th1 and exacerbate AT inflammation. In obesity, ATDCs have a lower capability to induce Tregs proliferation, while interaction with the Th2 subset is unknown. Separately, ATMs can uptake antigens inside the AT and induce proliferation of local effector CD4^+^ T cells into Th1 and/or Th17 subsets. 2. ATDCs partly regulate monocyte-derived ATM recruitment into AT, yet their interaction remains elusive. 3. Obese ATDCs fail to preserve their tolerogenic function in steady state to prevent preadipocyte differentiation and hyperplasia. Excess nutrition induces adipocyte hyperplasia, which decreases Wnt ligand and healthy dietary lipid secretion and leads to decreased Wnt/β-catenin signaling in cDC1s and inactive PPARγ in cDC2s, triggering an inflammatory state.

As a controller of innate and adaptive immunity, DC participates in the inflammatory function through the crosstalk with other cells. The diversity of AT resident cells facilitates the crosstalk between ATDCs and other key players that potentially regulate AT inflammation. Herein, we will discuss the interaction between ATDCs and T cells, ATMs, and other non-immune cells in response to environmental cues that potentially regulate AT inflammation, such as obesity, as summarized in Figure 2.

### 4.1. Regulator of Adipose Tissue Inflammation through T Cell Interaction

T cells play an important role in obesity-induced inflammation, and their numbers will increase in VAT obese mice and humans compared to a lean phenotype [63,64]. Interestingly, T cell expansion happens at early stages of obesity, when ATDCs are increasing and before the infiltration of ATMs to VAT [58,64,65]. Since naïve CD4^+^ T cells are activated after interaction with DC and differentiate into specific subtypes [66], crosstalk between ATDCs and T cells is considered as the primary mechanism to regulate AT inflammation.

Among diverse subsets of CD4^+^ cells, activation of T helper 1 (Th1) and T helper 17 (Th17) subsets has been implicated in obesity-induced adipose inflammation. Th1 and Th17 activation has a correlation with obesity, where IFNγ and IL-17 production are increased [30,57,58,67,68,69,70,71,72,73]. Consistent with the alteration of T cell subsets in obesity, obese ATDCs display higher capabilities to induce Th1 and Th17 activation seen in both human and mouse [30,52]. Moreover, ablation of AP function in DCs suppressed the obesity-induced CD4^+^ T cell increment in VAT [33]. These observations indicate that ATDCs mediate obesity-induced T cell activation. Furthermore, the IFNγ produced by Th1 will further activate DCs’ AP function to increase its MHC-II, in a feed-back loop, which would cause AT inflammation [58,65,68,70].

The T helper 2 (Th2) and Treg subsets are usually found in lean conditions and have anti-inflammatory properties, such as CD11c^−^ ATM activation [74,75]. In obesity, the percentage of Th2 and Treg is reduced as AT inflammation and insulin resistance progress, suggesting an important role of Th2 and Treg in AT homeostasis [72]. Ex vivo experiments revealed that obese ATDCs have lower ability to differentiate Treg [30,52]. Recently, it was reported that inactivation of *Ctnnb1* or *PPARγ* in ATDCs reduces Treg proliferation [38]. These findings indicate that tolerogenic effects of ATDCs through Th2/Treg differentiation would be essential to maintain AT homeostasis. 

Collectively, ATDCs interact with Th2/Treg in lean AT and potentially induce Th1 and Th17 proliferation in obesity. It remains unclear how ATDCs can play the tolerogenic or immunogenic functions depending on environmental cues or whether specific ATDC subsets have certain different functions.

### 4.2. Regulator of Adipose Tissue Inflammation by Macrophage Infiltration

Although ATDCs and ATMs share the same AP markers, their transcription profiles are unique, indicating that both are independent cells and they can crosstalk to each other in AT. The interaction between macrophages and DCs appears to be essential for the obesity-induced inflammation [12,31,48]. Stefanovic-Racic et al. found that *Flt3l*^−/−^ mice, which lack DCs, have lower ATM amounts, and the administration of DC or Flt3L promotes ATM infiltration [31]. Similarly, GM-CSF knockout mice showed lower ATM infiltration and accumulation under HFD, which contributes to amelioration of AT inflammation and insulin resistance [48]. In addition, a study used *Ccr7*^−/−^ mice, where lack of ATDCs was observed during lean and obese and demonstrated failure of ATM accumulation under obesity [12]. These findings suggest that ATDCs might regulate ATM function and accumulation, especially in obesity. However, as has been mentioned previously, these findings could not explain whether the effect is truly regulated by DC or not since other cells were also affected. It is not clear yet whether ATDCs and ATMs interact directly or indirectly through cytokine release, and whether activated-ATDCs could activate ATMs, or vice versa, or if each has an independent unrelated activation pathway. In the future, it is worth exploring how and which cells, whether ATMs and/or ATDCs, induce inflammation and insulin resistance in obesity.

### 4.3. Regulator of Adipose Tissue Homeostasis by Communication with Non-Immune Cells

Recently, immunometabolism has emerged as a major mechanism to DC immune regulation. In the AT environment, higher concentrations of lipid metabolites such as free fatty acid and cholesterol would mediate the interaction between adipocytes and ATDC, and then regulate AT inflammation. Conventional DCs can uptake the fatty acids released by adipocytes and use them as major fuel through oxidative phosphorylation or incorporate them in the membrane. Free fatty acids uptake by ATDCs will form lipid droplets (LD), which have important immunogenic properties, with high levels of LD promoting DC activation and antigen cross-presentation [76]. Not only CD4^+^ T cell activation, but cross-presentation activity was also induced by DC containing high level of LD (high-DC), as shown by increased CD8^+^ T cell activity. While high-DCs showed an immunogenic effect, low-DCs showed a tolerogenic effect with an increase of Tregs, reduced adipogenesis, and reduced endoplasmic reticulum (ER) stress. Tolerance can be achieved by increasing the low-DC/high-DC ratio [77]. The role of LD in ATDCs and ATMs and the regulation of obesity-induced inflammation still remain to be elucidated.

Interaction between ATDCs and preadipocytes is another contributor in AT homeostasis, especially in adipocyte hypertrophy and hyperplasia [47,78,79]. Absence of ATDCs in *Csf2*^−/−^ mice have increased adipogenesis, adiposity, and larger adipocytes. Further, not GM-CSF, but a DC conditioned medium enriched with MMP12 and fibronectin proteins could prevent preadipocyte to adipocyte differentiation. This was confirmed by upregulation of *Pref-1*, marker of the preadipocyte state [80], and downregulation of adipogenesis marker including *Pparγ*, *Cebpα*, and *Fabp4* [47]. Overall, these observations imply that the interaction between ATDCs and non-immune cells such as adipocytes and preadipocytes would be one of mechanism underlying the inflammation. 

## 5. Future Perspectives and Conclusions

Accumulating evidence clearly demonstrates that ATDCs are central players in the development of AT inflammation by interacting with several different types of cell types in AT, thereby initiating and regulating obesity-induced inflammation and insulin resistance. Recent studies showed that the imbalance between the tolerogenic and pro-inflammatory function of ATDCs is mainly attributed to obesity-induced inflammation. It has been also found that increasing number of ATDCs in the obese state is accompanied by different functional properties, including higher AP marker, to induce T cell activation and secretion of pro-inflammatory cytokines. Despite the rapid advances in immunometabolism, understanding mechanisms of how ATDCs contribute to obesity-associated diseases has still become an important issue, and further studies are needed to obtain future clinical and therapeutic designs against obesity-associated diseases. Obvious questions that remains are as follows: (1) what might be the antigens that are driving inflammation, (2) what triggers activate ATDCs during obesity, and (3) what are the regulatory mechanisms of specific ATDC subsets.

Identification of the specific presented-antigen would give promising ways to prevent obesity-induced inflammation by blocking AP itself or inhibiting antigen processing. The importance of antigen-presentation in the adipose inflammation is clear from studies that adipose tissue CD4^+^ T cells have restricted T cell repertoires, and the diversity is more restricted in the obese adipose tissue [81]. In addition, it is noteworthy that obesity promoted the expression of antigen presentation molecules such as MHC-II, and costimulatory molecule expression in visceral fat and deficiency of MHC-II in APC improved glucose homeostasis in obese mice [12,57]. However, the nature of the relevant antigen(s) is still entirely unknown in obesity, while several possible self-antigens, such as DNA damage, hypoxia, cell toxicity, and saturated lipid/cholesterol, have been proposed [82]. Recent advances in T cell repertoire analysis as well as new techniques to identify antigens for T cell receptors may enable a successful search for antigens in obesity in a systematic way.

In peripheral tissues, resident DCs are activated directly by conserved pathogen molecules and indirectly by inflammatory mediators produced by other cell types that recognize such molecules. Interactions with CD4^+^ and CD8^+^ T cells can also induce DC activation. Although the activation of ATDCs in obesity is well known, it remains unelucidated why and how ATDCs become activated, and whether it would be due to other cell-derived factors or interaction with T cells. One study showed that HFD induced the gut microbiota-derived LPS alteration and increased the uptake of LPS to adipose tissue [83], indicating gut-microbiota derived pathogen might be a metabolic trigger to activate ATDCs during obesity. A recent study also demonstrated that modulating checkpoint co-inhibitory interactions have been successful in animal models of obesity-associated inflammation and metabolic dysfunctions [84], suggesting that interactions through co-stimulatory or co-inhibitory molecules would be another factor in the activation of ATDCs in obesity.

ATDCs are heterogenous populations with different subsets that can function as either tolerogenic or inflammatory actions [16]. Considering the diversity of ATDC subsets, comprehensive studies about interaction of specific ATDC subsets with other immune or non-immune cells would give greater knowledge. However, current studies about ATDCs in obesity have been limited due to the lack of animal models. One potential caveat of global DC-deficient models including *Flt3l*^−/−^, *Csf2*^−/−^, and *Ccr7*^−/−^ is the unavoidable defects to reflect the developmental and physiological consequences of DC deficiency in steady state. Another caveat of current animal models is lack of specificity of DC. Thus, a clear inducible model that could ablate ATDCs only during obesity progression is needed to elucidate specific ATDC actions. As a means to specifically deplete cDCs, a cDNA encoding human DTR was introduced into the 3′ untranslated region of the mouse *Zbtb46* (also called zDC) gene. Unlike the previously characterized CD11c-DTR mice, non-cDCs such as pDCs, monocytes, macrophages, and NK cells were spared after DT administration [19]. The use of these systems will most certainly help clarify the role of cDCs in the initiation and development of obesity-associated AT inflammation and bring us a step closer to finding better therapeutic strategies. It is also needed to generate new animal models to investigate the function of specific DC subsets.

Based on the central role of ATDCs in the obesity-induced inflammation, ATDCs are now considered as the potential targets to modulate metabolic diseases. Targeting to all the DCs or even general molecules on ATDCs is inappropriate, since global depletion of DCs is very deleterious [85,86]. Rather, therapeutic interventions may target antigen or co-stimulatory/coinhibitory molecules, which are selectively acting under the pro-inflammatory state in obesity. Antigen-specific therapy may hold promise in the treatment of the metabolic syndrome. Future research should elucidate antigens during obesity and determine the best mechanism to target adaptive immunity to improve metabolic function in obesity. Further investigation and integration of these fields will be necessary to overcome obstacles and to open new approaches to improve the promising therapeutic option of ATDCs for the future.

## Data Availability

Not applicable.

## Abbreviations

AP	Antigen Presentation
APC	Antigen-Presenting Cell
AT	Adipose Tissue
ATDC	Adipose Tissue Dendritic Cell
ATM	Adipose Tissue Macrophage
BAT	Brown Adipose Tissue
BATF	Basic Leucine Zipper ATF (Activating Transcription Factor)-like
BMI	Body Mass Index
CCL	C-motif Chemokine Ligand
CCR	C-C Chemokine Receptor
CD	Cluster of Differentiation
cDC	Conventional DC
CLEC	C-type Lectin Domain
CLS	Crown Like Structures
CLV	Collecting Lymphatic Vessel
DC	Dendritic Cell
DIO	Diet-induced Obesity
DT	Diphtheria Toxin
DTR	Diphtheria Toxin Receptor
ER	Endoplasmic Reticulum
FMS	Feline McDonough Sarcoma
FLT3	FMS-like Tyrosine Kinase
FLT3L	FMS-like Tyrosine Kinase Ligand
FN	Fibronectin
GM-CSF	Granulocyte Macrophage Colony Stimulating Factor
HFD	High Fat Diet
HSPC	Hematopoietic Stem and Progenitor Cell
IL	Interleukin
IRF	Interferon Regulatory Factor
ITGAX	Integrin Alpha X
MAPK	Mitogen-Activated Protein Kinase
MerTK	MER Proto-Oncogene, Tyrosine Kinase
MHC	Major Histocompatibility Complex
MMP	Matrix Metallopeptidase
moDC	Monocyte-derived DC
ND	Normal Diet
pDC	Plasmacytoid DC
SAT	Subcutaneous Adipose Tissue
SLO	Secondary Lymphoid Organ
TCR	T Cell Receptor
TLR	Toll-like Receptor
TREM	Triggering Receptor Expressed on Myeloid Cell
UCP	Uncoupling Protein
VAT	Visceral Adipose Tissue
ZBTB	Zinc Finger and BTB (Broad-Complex, Tramtrack and Bric a Brac)-domain
